# Finite element evaluation of an American football helmet featuring liquid shock absorbers for protecting against concussive and subconcussive head impacts

**DOI:** 10.3389/fbioe.2023.1160387

**Published:** 2023-06-09

**Authors:** Nicholas J. Cecchi, Hossein Vahid Alizadeh, Yuzhe Liu, David B. Camarillo

**Affiliations:** ^1^ Department of Bioengineering, Stanford University, Stanford, CA, United States; ^2^ School of Biological Science and Medical Engineering, Beihang University, Beijing, China; ^3^ Savior Brain, Inc., Arlington, VA, United States

**Keywords:** concussion, brain injury, hydraulic shock absorber, helmet, brain strain, finite element modeling

## Abstract

**Introduction:** Concern has grown over the potential long-term effects of repeated head impacts and concussions in American football. Recent advances in impact engineering have yielded the development of soft, collapsible, liquid shock absorbers, which have demonstrated the ability to dramatically attenuate impact forces relative to existing helmet shock absorbers.

**Methods:** To further explore how liquid shock absorbers can improve the efficacy of an American football helmet, we developed and optimized a finite element (FE) helmet model including 21 liquid shock absorbers spread out throughout the helmet. Using FE models of an anthropomorphic test headform and linear impactor, a previously published impact test protocol representative of concussive National Football League impacts (six impact locations, three velocities) was performed on the liquid FE helmet model and four existing FE helmet models. We also evaluated the helmets at three lower impact velocities representative of subconcussive football impacts. Head kinematics were recorded for each impact and used to compute the Head Acceleration Response Metric (HARM), a metric factoring in both linear and angular head kinematics and used to evaluate helmet performance. The head kinematics were also input to a FE model of the head and brain to calculate the resulting brain strain from each impact.

**Results:** The liquid helmet model yielded the lowest value of HARM at 33 of the 36 impact conditions, offering an average 33.0% (range: −37.5% to 56.0%) and 32.0% (range: −2.2% to 50.5%) reduction over the existing helmet models at each impact condition in the subconcussive and concussive tests, respectively. The liquid helmet had a Helmet Performance Score (calculated using a summation of HARM values weighted based on injury incidence data) of 0.71, compared to scores ranging from 1.07 – 1.21 from the other four FE helmet models. Resulting brain strains were also lower in the liquid helmet.

**Discussion:** The results of this study demonstrate the promising ability of liquid shock absorbers to improve helmet safety performance and encourage the development of physical prototypes of helmets featuring this technology. The implications of the observed reductions on brain injury risk are discussed.

## Introduction

Sports and recreation can present a considerable risk of brain injury, with an estimated 1.6 to 3.8 million mild traumatic brain injuries, or concussions, occurring annually in the United States as a result of participation in these activities (Langlois et al., 2006). American football, in particular, presents a significant risk for concussion relative to other sports (Lincoln et al., 2011; Kerr et al., 2019). At the elite level of play (i.e., the National Football League (NFL)), one study found that the risk of concussion between the 2015 and 2019 seasons was 7.4% per player per season on average (Mack et al., 2021). In another study, single season concussion risks in youth, high school, and collegiate American football were found to be as high as 3.53, 9.98, and 5.54% per player per season, respectively (Dompier et al., 2015). Sport-related concussion can be followed by physical, behavioral, somatic, and cognitive symptoms (McCrory et al., 2017) that can last from the span of days, weeks, or even months as post-concussion syndrome (Broshek et al., 2015). Furthermore, while more research is required, several studies have found associations between concussion history and later life cognitive impairment and neurodegenerative disease development (Manley et al., 2017). Aside from the health risks, concussion can also have negative effects on athlete performance, career longevity, and salary earnings for professional American football players (Navarro et al., 2017). Therefore, concussion prevention remains a prevalent focus of sport policy changes and protective equipment innovations in the sport.

Even in the absence of diagnosed concussions, American football athletes are prone to sustaining repeated, subconcussive head impacts during their regular practices and competitions (Karton et al., 2020; Cecchi et al., 2021; Choi et al., 2022; Marks et al., 2022). Subconcussive head impacts, broadly, are those which are not of great enough magnitude to result in a clinically diagnosed concussion, but may still contribute to detectable short or long-term health effects (Mainwaring et al., 2018). The accumulation of subconcussive head impacts has been associated with both acute and chronic neurological consequences (Mainwaring et al., 2018), including development of neurodegenerative disease (Russell et al., 2021). Further, in American football players specifically, subconcussive head impacts have been associated with various indicators of brain changes, including those observed via imaging studies (Davenport et al., 2016; Foss et al., 2019), changes in oculomotor function (Joseph et al., 2019), and changes in levels of serum biomarkers of brain injury (Joseph et al., 2018). Therefore, attenuation of subconcussive impacts, in addition to concussion prevention, has become a recent priority in improving long term athlete brain health.

In an effort to reduce the risk of head and brain injury, protective helmets are required to be worn at all levels of American football competition. Helmets have evolved dramatically since their first introduction to the sport (Levy et al., 2004; Viano and Halstead, 2012) and presently consist of a variety of shock absorbing technologies with differing mechanisms for energy absorption and force attenuation (Hoshizaki et al., 2014; Dymek et al., 2022). Notably, some modern technologies include viscoelastic foams, buckling beams and structures, gas chambers, and 3D printed lattices. Helmet impact velocities range considerably in American football (Bailey et al., 2020a); to best protect an athlete from concussive and subconcussive head impacts, a helmet should be designed such that it can meaningfully attenuate impacts of both low and high velocities. However, current testing standards and rankings for helmets place emphasis on the ability of helmets to attenuate the magnitude of impacts associated with diagnosed concussions or more serious injuries (i.e., skull fractures) (Rowson and Duma, 2011; Bailey et al., 2020b; NOCSAE, 2021). While it appears that progress has been made in reducing the incidence of concussion and skull fractures as a result of some of these testing protocols (Viano and Halstead, 2012; Bailey et al., 2020a), a concern exists that these protocols may result in helmets only being optimized for peak performance upon high velocity impacts representative of concussions. An ideal helmet shock absorber would perform optimally across the entire range of impact velocities that an athlete is exposed to, inclusive of both concussive and subconcussive impacts.

An ideal shock absorber performs fundamentally different from existing foams, solid structures, and gas-based technologies. In practice, many of these existing technologies exert a force based on the amount they are compressed, leaving them prone to high spikes in reaction force when they reach maximum compression or “bottom out” (Fanton et al., 2020). The impact force profile of an ideal shock absorber, on the other hand, displaces through its full stroke at a constant force, which scales based on the speed and energy of an impact speed (Baumeister et al., 1997; Spinelli et al., 2018; Vahid Alizadeh et al., 2022). Research has suggested that, in the context of a protective helmet, a constant force profile is optimal for preventing brain injury (Vahid Alizadeh et al., 2021). While gases and solids have yet to achieve such a response that is capable of scaling with impact velocity, prototype and computational models of liquid shock absorbers have demonstrated promising results on achieving a force profile near to that of an ideal shock absorber (Fanton et al., 2020; Vahid Alizadeh et al., 2021; Vahid Alizadeh et al., 2022).

In addition to being used for modeling of shock absorbing technologies, finite element (FE) modeling has been used to investigate brain injury risk and advance the state-of-the-art of helmet technology (Dymek et al., 2022). Validated FE models of helmets and laboratory testing equipment have been developed to simulate laboratory impact tests of helmets (Bustamante et al., 2019; Corrales et al., 2020; Decker et al., 2020; Giudice et al., 2019; Giudice et al., 2020; Vahid Alizadeh et al., 2021), enabling researchers and helmet manufacturers to rapidly iterate designs and estimate the performance of helmets before engaging in large scale manufacturing. Further, the head kinematics resulting from these simulated impacts can be used as input to validated FE models of the human head and brain to estimate the brain strain resulting from impacts (Madhukar and Ostoja-Starzewski, 2019). Use of these computational tools can reduce cost, time, and difficulty in bringing new helmet technologies to market and aid in identifying the most promising technologies to prevent brain injury. In a previous study, an FE model of a helmet design utilizing theoretically idealized liquid shock absorber elements suggested a dramatic reduction in concussion risk was possible with this technology, but a method of manufacturing such a helmet was not explored and performance under lower velocity impacts was not studied (Vahid Alizadeh et al., 2021). Development of further, more advanced FE models of liquid shock absorbing technology could enable full helmet systems to more quickly reach consumers and reduce injury risk among the American football athlete population.

The objective of this research study was to use FE modeling to investigate the potential for a liquid-based shock absorber to improve the ability of American football helmets to attenuate the severity of both concussive and subconcussive head impacts in American football. To do this, we developed a helmet assembly with liquid-based shock absorbers integrated throughout it that would be feasible for physical re-creation. Further, we used a FE model of the human head and brain to determine not only the effect of this technology on brain injury risk metrics based on head kinematics, but also on the resulting brain strain from the simulated impacts.

## Materials and methods

### Liquid shock absorber design

**FIGURE 1:**
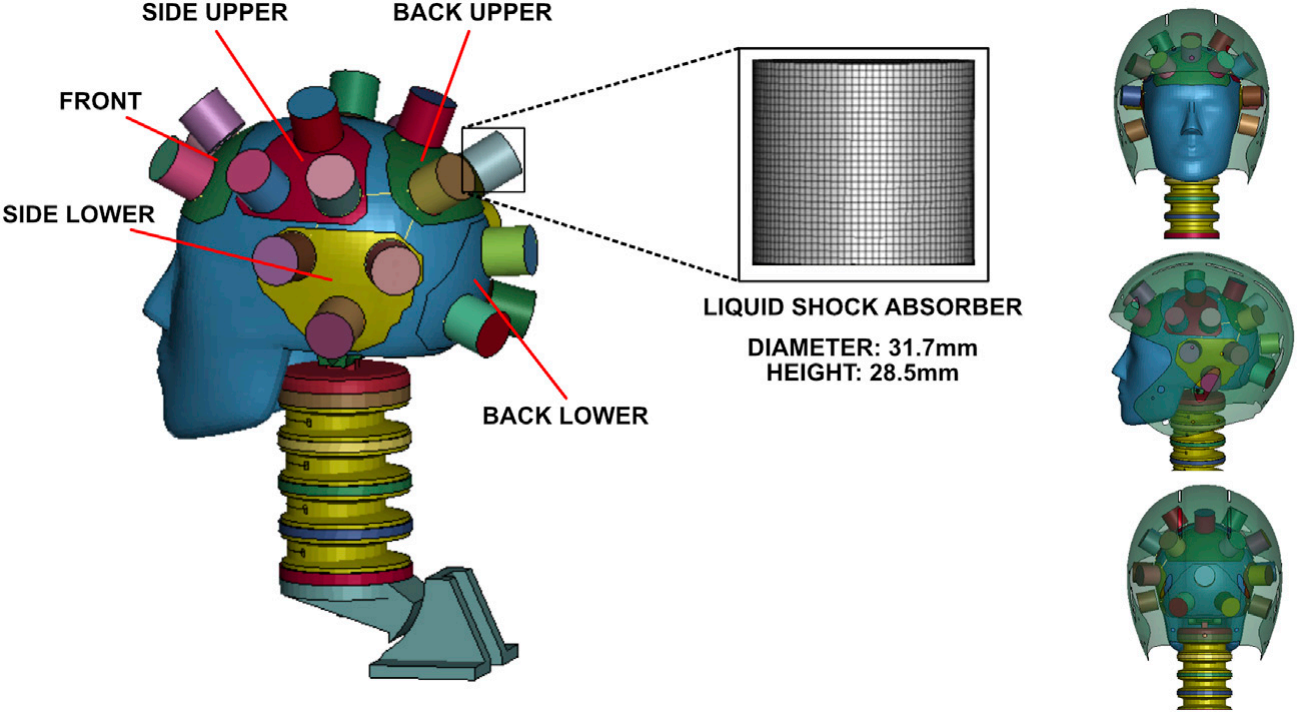
Arrangement of liquid shock absorbers in helmet finite element model.

The liquid shock absorber utilized throughout this study was modeled as a cylinder that can decelerate an impact mass in its axial direction **(** Figure 1 **)**, which was inspired by the cylindrical liquid shock absorber originally modeled by Vahid Alizadeh et al. (2022). In an impact, the top surface of the cylinder is pressed downwards, and the liquid inside is forced to flow through the orifice. The liquid passing through the orifice yields a pressure drop before and after the orifice, defined by Eq. 1:

$$\Delta p=\frac{\rho Q^{2}}{2C_{d}^{2}A_{o}^{2}}$$ (1)

Where ρ is the fluid density, Q is the volumetric flow rate, $C_{d}$ is the orifice discharge coefficient, $A_{o}$ is the orifice area, and Δp is the pressure differential, ${p-p}_{o}$ , where p is the fluid pressure inside the liquid shock absorber and $p_{o}$ is the atmospheric pressure downstream of the orifice. Because the pressure at the outlet of the orifice is the same as the atmospheric pressure, the inner pressure of the shock absorber is higher, which can yield a resultant force to decelerate an impact mass. Furthermore, it should be noted that the pressure drop is also decided by the orifice area, which indicates that the resultant force can be easily tuned by modifying the orifice area.

A plain-weave high-strength fabric is used as the side wall of the shock absorber to constrain localized bulging and avoid rupturing of the side wall during impact while also allowing the shock absorber to be fully compressed. Carbon fiber shells are used for the top and bottom end caps of the cylinder to provide lightweight, rigid constraints at both ends of the shock absorber, enabling it to hold its cylindrical shape during impact. Water is used to fill the shock absorber and dissipate impact energy. The fabric side wall and carbon fiber end caps are nonporous, and the connection between the fabric and the end caps are modeled as sealed connections; therefore, fluid can only be discharged through the orifice. The orifice area is tuned to optimize helmet performance, which will be introduced later.

The height of the shock absorber was set to 28.5 mm, which was chosen to ensure that it could fit within the space between the scalp of the head and the shell of an American football helmet. To avoid axial buckling during impact, a larger diameter of the cylindrical shock absorber is better. However, smaller diameters are also favorable, considering that the shock absorbers are distributed across the scalp, which is a curved surface. Therefore, based on these two considerations, a diameter slightly larger than the height (31.7 mm) was adopted. A thickness of 0.6 mm was adopted for the side wall fabric, and a thickness of 2 mm was used for both end caps.

The liquid shock absorber was modeled as quadrilateral shell elements in LS-DYNA. Because the fabric can be bent freely, the side wall of the shock absorber was modeled by fully integrated Belytschko-Tsay membrane shell elements, which only have one integration point in the thickness direction and neglect the bending effect. Furthermore, multiple material angles were included to represent the different weaving directions of the fibers. *MAT_FABRIC was used to model the fabric material, which has a density of 777 kg/m^3^, longitudinal modulus of 76 GPa, transverse modulus of 0.6 GPa, and shearing modulus of 0.8 Gpa (Vahid Alizadeh et al., 2022). The side wall was meshed by 1,292 elements with an average length of 1.5 mm. The top and bottom caps were modeled by Belytschko-Tsay shell elements with two nodes in the thickness direction. The *MAT_ELASTIC was adopted to model the material, which has a density of 2000 kg/m^3^, Young’s modulus of 200 GPa, and Poisson ratio of 0.23. A cap was meshed by 292 elements with an average length of 1.5 mm. The side wall and end caps were connected by sharing nodes.

The effect of the orifice was modeled by *AIRBAG_LINEAR_FLUID_ID, which exerted pressure on the internal surface of the shock absorber according to the mass rate of the flow (Eq. 2):

$${ṁ}_{o}=sign\left(\Delta p\right)C_{d}A_{o}{\left[2\rho \Delta p\right]}^{1/2}$$ (2)

As mentioned earlier, Δp is the pressure differential, ${p-p}_{o}$ , where p is the pressure inside the shock absorber and $p_{o}$ is the atmospheric pressure downstream of the orifice, or the back pressure. This back pressure plays the role of returning the fluid to the shock absorber after an impact has occurred. Upon the completion of impact loading, the pressure inside the shock absorber, or the upstream pressure, is lower than the back pressure and this negative pressure differential returns the fluid back into the shock absorber.

An internal incompressible fluid with water properties was used as the liquid inside the shock absorber (bulk modulus is 2.2 Gpa, density is 1,000 kg/m^3^), and a discharge coefficient of 0.7 was used (Fanton et al., 2020; Vahid Alizadeh et al., 2022). The water was modeled as a control volume and no mesh was associated with the fluid part, but the inertia of the liquid was included. The orifice area is a key parameter determining the impact dissipation performance of the helmet. Orifice areas ranging from 10 to 200 mm^2^, in increments of 5 mm^2^, were adopted homogeneously for all shock absorbers in the helmet model and subjected to the NFL helmet test protocol (Bailey et al., 2020b, details of this test protocol are described in the following *Helmet Performance Testing* section). The Helmet Performance Score (HPS, see the calculation of HPS in the following *Helmet Performance Testing* section) was calculated for every orifice area to represent the risk of brain injury when the player is wearing that helmet. The HPS was found to increase when the orifice was too small (<40 mm^2^) or too large (>70 mm^2^) and remained relatively constant within these bounds (Supplementary Figure S1). Therefore, we adopted an orifice area of 59 mm^2^, which corresponded the lowest level of HPS when evaluating HPS in orifice area increments of 1 mm^2^ between 40 mm^2^ and 80 mm^2^ (Supplementary Figure S2). It should be noted that small variations could be observed between simulations of the same orifice area when evaluated in the two separate optimizations, likely due to small numerical errors.

### Helmet assembly

A previously validated, open-sourced FE model for the Vicis Zero1 helmet (Giudice et al., 2020) was used as the base model for our full helmet assembly, and we modified the VICIS Zero1 helmet to test the helmet performance improvement afforded by integration of liquid shock absorbers. The original Vicis Zero1 dissipates impact energy by the buckling of elastic beams, which were removed. The material of the outer helmet shell was changed to a carbon fiber (with the same properties as the shock absorber end caps). The carbon fiber was substantially stiffer than the original helmet material and, therefore, could involve more shock absorbers during the impact due to its limited local deformation. The remaining parts of the helmet were kept the same as the original helmet model: the original facemask, chin pad, and chin strap were adopted in the liquid helmet model.

To integrate the cylindrical liquid shock absorbers into the full helmet system, we first fixed three shock absorbers onto a triangular bottom shell made of 2 mm thick carbon fiber (forming a “tripad”). To fix a shock absorber on the bottom shell, a set of 8 nodes evenly distributed about the circumference of the bottom cap of the shock absorber were rigidly connected to 8 nearby nodes in the bottom shell by *CONSTRAINED_NODAL_RIGID_BODY, which constrains every degree of freedom of nodes to be the same. We used seven tripad assemblies spread out throughout the helmet to protect the head from impacts at various locations: one at the front, one at the lower back, one at the upper back, and a lower and upper side tripad on each side of the helmet (Figure 1). This resulted in 21 liquid shock absorbers integrated throughout the helmet. For each of the tripads, the geometry of the bottom carbon fiber shell was decided by the skull, such that the surface of the Hybrid III ATD headform was extracted and cut into the triangular shells. Then, the triangular shells were offset against the skull as the bottom shell for the tripads. The exact shape of the shock absorber tripad shell and the location of shock absorbers within each tripad assembly varied such that shock absorbers would be distributed evenly around the skull and symmetrically with respect to the sagittal plane of the head. Finally, adjacent tripads were connected by elastic beams with a cross-sectional area of 19.6 mm^2^. A relatively soft material (Young’s modulus: 10 GPa, density: 1,631 kg/m^3^, Poisson ratio: 0.4) was used for the elastic beams, allowing the tripads to shift for better fitting to the head. The total mass of the helmet was determined to be 2.03 kg.

### Helmet Performance Testing

The single-precision solver of LS-DYNA (ls-dyna_smp_s_r1010_x64_redhat5_ifort160) was used to perform all helmet simulation experiments. The efficacy of the liquid helmet was tested with a validated, open source model of a linear impactor and 50th percentile male Hybrid III anthropomorphic test device headform (Giudice et al., 2019). The model used was made to replicate the equipment used in the NFL’s Helmet Test Protocol (Bailey et al., 2020a). In these tests, a Hybrid III headform is equipped with a helmet and is impacted at a controlled speed by a ram. The impactor consists of a hard end cap and a soft foam, which is meant to represent another helmeted head. The total mass of the impactor is 15.6 kg. The headform was connected to a 50th percentile male Hybrid III neck, which was fixed to a plate that can only slide freely in the direction of the impact ram. The relative location between the impactor and headform, as well as the angles of the headform, could be adjusted to achieve different impact locations and directions. A set of gyroscopes and accelerometers were modeled at the center of the headform to measure the six-degree-of-freedom head kinematics (i.e., angular velocities and linear accelerations) resulting from impacts. The whole testing process has been modeled in FE (Giudice et al., 2019) and is also used in evaluating physical football helmets (Bailey et al., 2020b).

**FIGURE 2:**
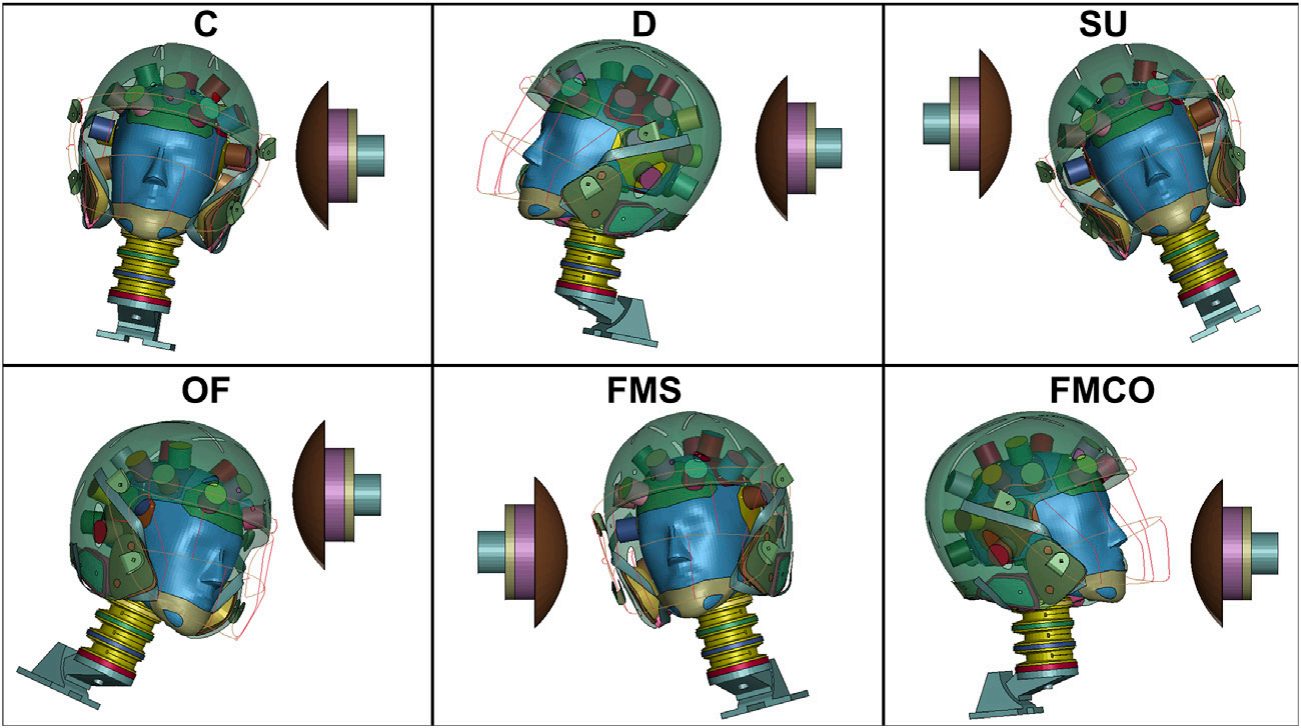
Impact testing locations. C: Side, D: Oblique Rear, SU: Side Upper, OF: Oblique Front, FMS: Facemask Side, FMCO: Facemask Central Oblique.

**FIGURE 3:**
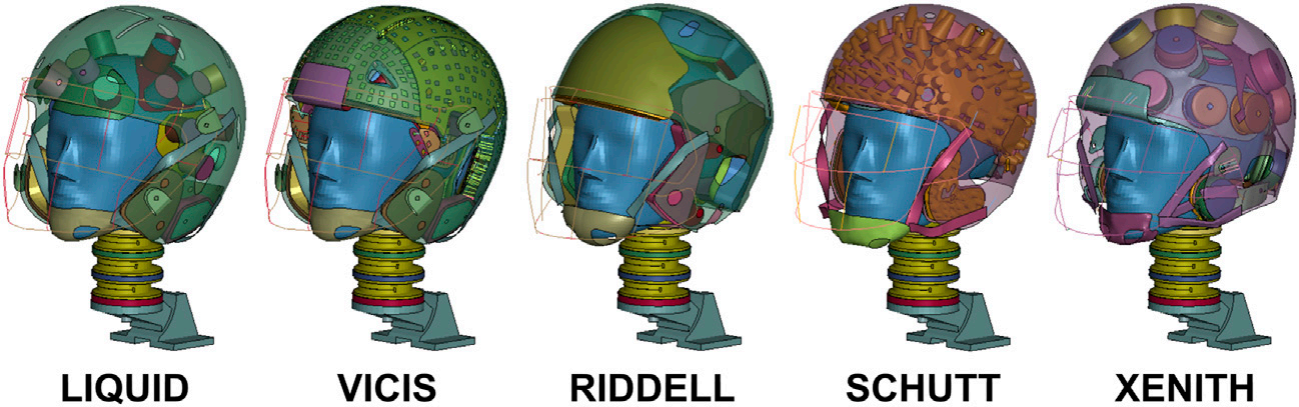
Finite element models of American football helmets tested in simulation. Each model is fit to the 50th percentile male Hybrid III head and neck finite element model. Helmet shells are set to 50% transparency for visualization of interior shock absorbing technology.

Testing was completed according to the NFL’s Helmet Test Protocol. In this test protocol, there are six impact locations: Oblique Front (OF), Side (C), Side Upper (SU), Oblique Rear (D), Facemask Side (FMS), and Facemask Central Oblique (FMCO) **(** Figure 2 **)** and three impact speeds: 5.5, 7.4, 9.3 m/s (Bailey et al., 2020a). Considering that this test protocol was developed primarily to replicate concussive head impacts, three lower speeds (1.6, 3.4, 5.0 m/s) representative of average impact velocities (inclusive of injurious and noninjurious impacts) (Bailey et al., 2020b) were also included for testing, and are referred to as subconcussive impact tests. In addition to the liquid helmet model designed in this study, four previously created, open-source football helmet FE models were also tested under the same impact conditions, including: the Riddell Revolution Speed Classic, the Vicis Zero1 (Giudice et al., 2020), the Schutt Air XP Pro (Decker et al., 2020), and the Xenith X2E (Corrales et al., 2020) **(** Figure 3 **)**. The masses of each of these helmets were measured in LS-DYNA as 1.86, 2.12, 1.71, and 1.74 kg, respectively.

Similar to the NFL Helmet Test Protocol, Head Injury Criterion (HIC) (Versace, 1971) and DAMAGE (Gabler et al., 2019) and HARM (Bailey et al., 2020a) were calculated for all impact conditions. These metrics were calculated using the head kinematics filtered with a cut-off frequency of 300 Hz. In three impact cases, the VICIS helmet model erroneously terminated prior to the completion of the impact event and therefore these cases have lower values of injury risk metrics than if the simulation had completed in full; these cases are labeled in the appropriate figures. The percentage reduction in HARM afforded by the liquid helmet relative to each existing helmet model was calculated. A Helmet Performance Score was calculated for each helmet using a weighted sum of HARM values from the concussive impact tests, with each HARM value being weighted according to the NFL’s Helmet Test Protocol (Bailey et al., 2020b).

**FIGURE 4:**
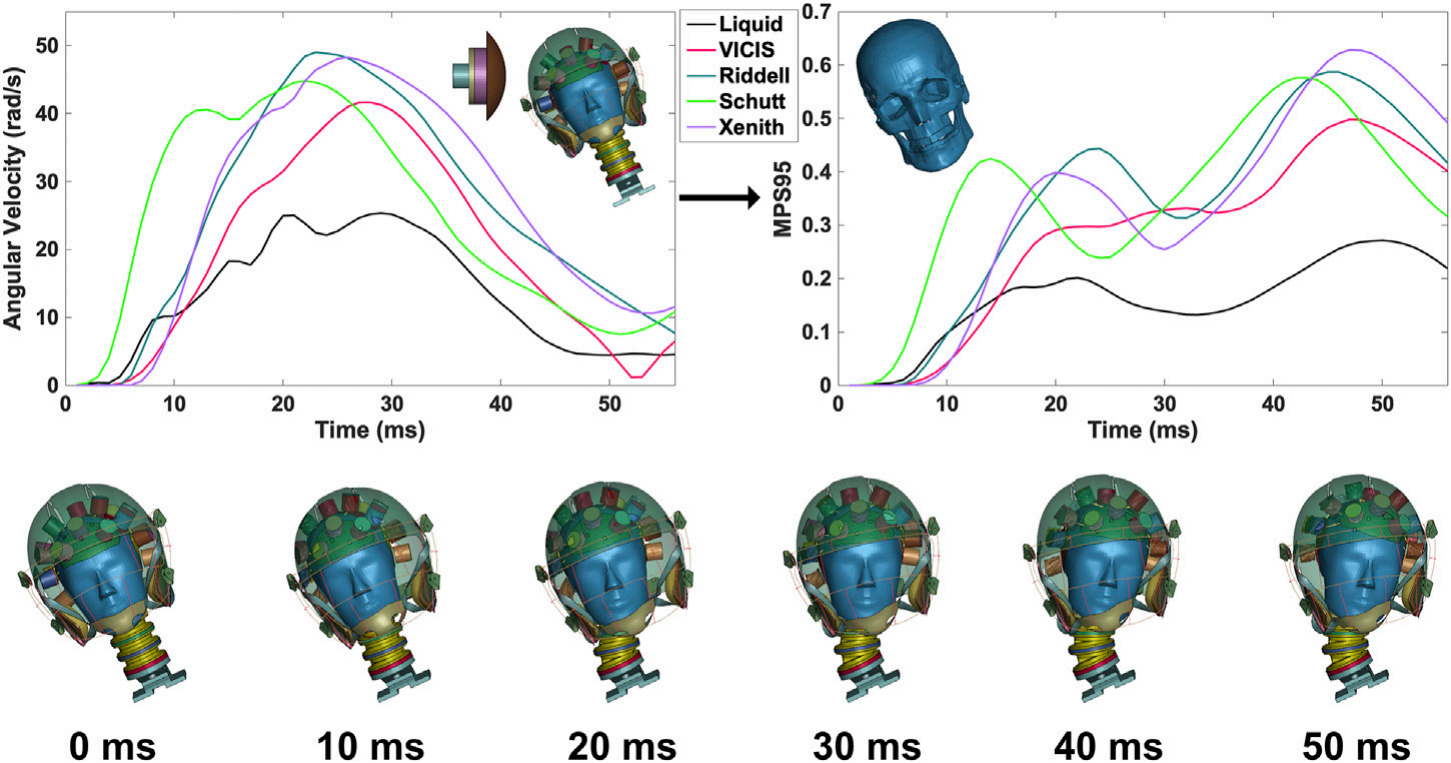
Example of angular velocity time histories from helmet impact simulations (top left) that are used as input to the GHBMC head and brain model to compute 95th percentile maximum principal strains (top right). The headform and helmet assembly at various time points during the impact process are shown (bottom). All data shown is from the Side Upper impact location at 9.3 m/s.

In addition to these kinematics-based metrics, we also calculated the expected strain on the brain tissue to show how different helmet technologies affect the human brain. In this study, the Global Human Body Model Consortium (GHBMC) head and brain model was used (Mao et al., 2013). The double-precision solver of LS-DYNA (ls-dyna_smp_d_r1010_x64_redhat5_ifort160) was used to perform all brain strain simulations. Its skull was modified to act as a rigid body. The filtered head kinematics were assigned to the rigid skull as the loading to deform brain tissue, and the 95th percentile maximum principal strain (MPS95) across the whole brain was calculated for every time point of each simulation **(** Figure 4 **)**. The 95th percentile of maximum principal strain across the whole brain was used instead of the maximum principal strain to avoid the influence of extremely high values caused by numerical errors. Then, peak values of MPS95 across the full time history of each impact were recorded to represent the severity of brain deformation.

## Results

**FIGURE 5:**
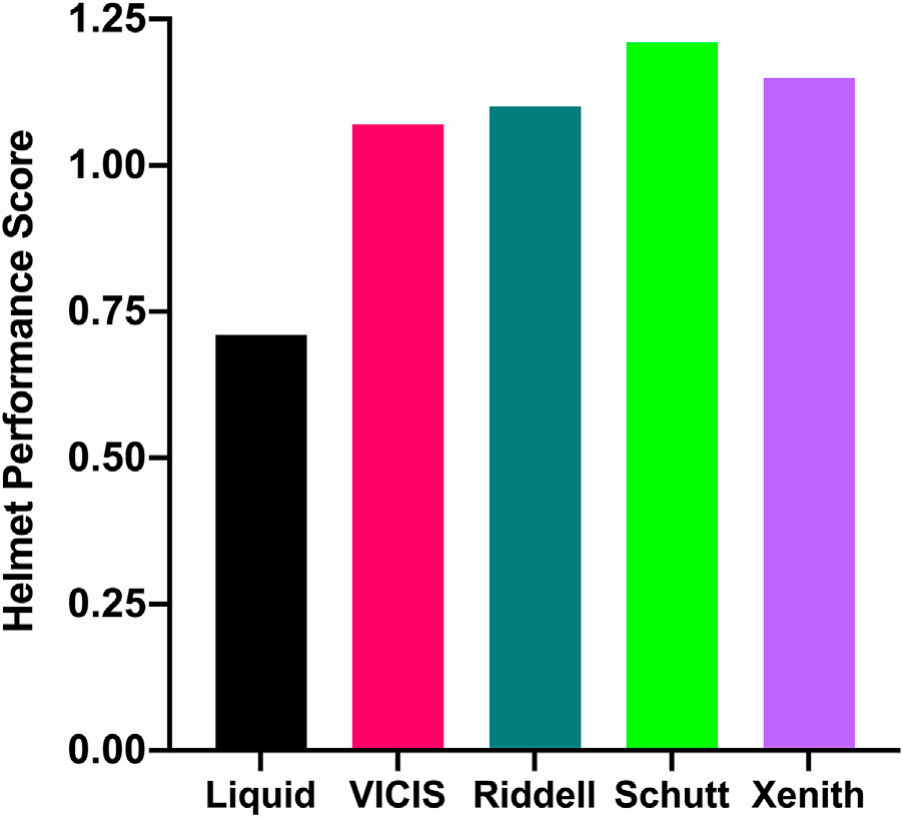
Helmet Performance Scores calculated from the NFL Helmet Test Protocol’s concussive impact tests for each of the five helmets tested.

**FIGURE 6:**
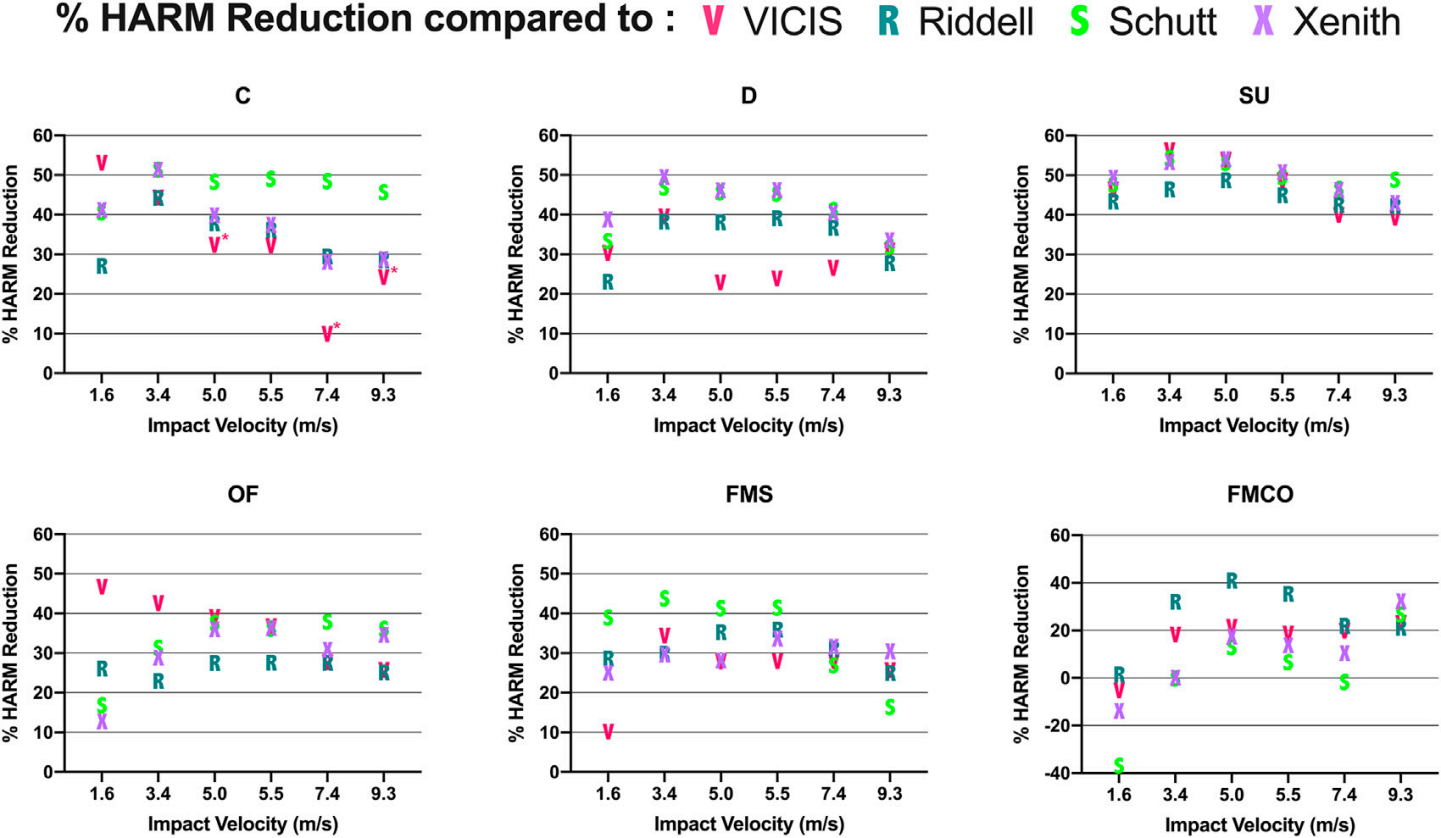
Reductions in HARM values afforded by the liquid helmet, relative to each existing helmet model at each impact condition. Positive values indicate that the liquid helmet had a lower value of HARM relative to a given helmet, while negative values indicated that the liquid helmet had a higher value of HARM. Asterisks denote instances in which impact simulations of the VICIS helmet model erroneously terminated prior to the completion of the impact event. C: Side, D: Oblique Rear, SU: Side Upper, OF: Oblique Front, FMS: Facemask Side, FMCO: Facemask Central Oblique.

Overall, the liquid helmet yielded the lowest Helmet Performance Score (0.71), compared to the other four helmet models (VICIS = 1.07, Riddell = 1.10, Schutt = 1.21, Xenith = 1.15) **(** Figure 5 **)**. The liquid helmet yielded the lowest value of HARM at 33 of the 36 tested impact conditions, with 3 velocities at the FMCO location being the only conditions in which the liquid design did not outperform all of the existing helmet models. Relative to all helmet models, HARM reductions afforded by the liquid helmet averaged 32.0% (range: -2.2%–50.5%) at concussive velocity impact cases and averaged 33.0% (range: -37.5%–56.0%) at subconcussive velocity impact cases **(** Figure 6 **)**.

**FIGURE 7:**
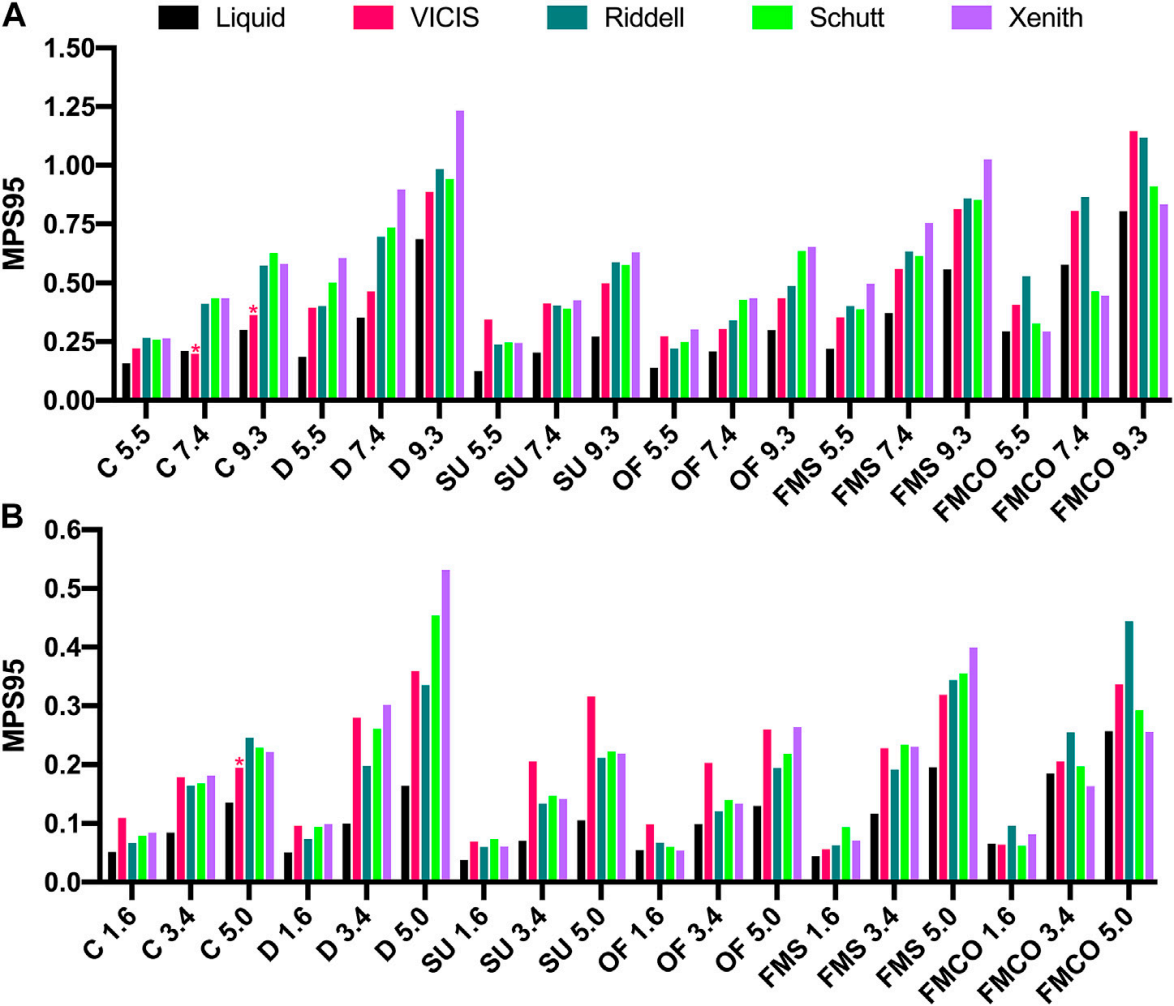
95th percentile maximum principal strain calculated via the GHMBC head and brain model for **(A)** concussive and **(B)** subconcussive impacts. Asterisks denote instances in which impact simulations of the VICIS helmet model erroneously terminated prior to the completion of the impact event. C: Side, D: Oblique Rear, SU: Side Upper, OF: Oblique Front, FMS: Facemask Side, FMCO: Facemask Central Oblique.

MPS95 values for each helmet at each impact condition can be found in Figure 7A, 7B, for the concussive and subconcussive impact tests, respectively. HIC, DAMAGE, and HARM values for each helmet at each impact condition can be found in Supplementary Figures S3, S4, for the concussive and subconcussive impact tests, respectively.

## Discussion

In this study, we developed a FE model of a helmet featuring liquid shock absorbing technology with the goal of attenuating the severity of concussive and subconcussive head impacts in American football. Compared with four existing FE helmet models, our simulation results demonstrate that liquid shock absorbers have the potential to provide a considerable reduction in kinematics-based brain injury criteria across a wide range of impact velocities and impact locations. Using a FE model of the human head and brain to investigate the effects of the liquid helmet on resulting brain strains also suggested that dramatic attenuations of impact severity could be achieved, which could be meaningful when considering brain injury risk.

The liquid helmet model we developed yielded a substantially lower value of Helmet Performance Score than any of the other FE helmet models tested, reducing Helmet Performance Score by approximately 34% compared to the best previously existing FE helmet model. Helmet Performance Score is the primary metric by which helmets are ranked for safety according to the NFL (Bailey et al., 2020a), and it is used to create an annual ranking of helmets that is published to all NFL athletes and the general public each year. Helmet Performance Score is an aggregate metric that combines the performance of helmets at various impact conditions by weighting their value of HARM based on relative risk of concussion incidence and then summing them together. The highest weighted impact location in the Helmet Performance Score is the Side Upper location, due to its high incidence of concussion in elite game play (Lessley et al., 2018). The liquid helmet model performed particularly well at this location, offering improvements in HARM by a range of 39%–50% across all concussive impact velocities, thus driving a lower Helmet Performance Score. However, the liquid helmet did not seem to compromise performance at concussive velocities at any location as a result of this highly improved location, offering HARM reductions at all but one of the concussive impact conditions (FMCO 7.4 m/s, 2% HARM increase relative to the Schutt helmet). Helmet Performance Score, and the HARM, HIC, and DAMAGE values from which it is derived, may be meaningful metrics to consider when attempting to reduce injury incidence on the field; a recent study found that Helmet Performance Score calculated from laboratory tests of helmets was associated with on-field concussion incidence in NFL game play (Bailey et al., 2020b). According to the data provided in that study, a Helmet Performance Score of 0.71 would represent a substantial improvement upon existing helmets. It should be noted that Helmet Performance Score is designed specifically for the NFL, and further investigation is warranted to determine how our findings would translate to a metric like Helmet Performance Score that was made for the youth, high school, and collegiate levels of game play. Nonetheless, other studies have found that helmets yielding improved laboratory performance (as defined by reductions in kinematics-based brain injury risk metrics) have been associated with reductions in on-field concussion incidence (Collins et al., 2006; Rowson et al., 2014) and preservation of brain white matter (Diekfuss et al., 2021) at levels of game play lower than the elite, professional level.

In American football, the number of subconcussive impacts athletes sustain far outweighs the number of diagnosed concussions in the sport (Cecchi et al., 2021; Choi et al., 2022; Marks et al., 2022). However, despite the prevalence of low velocity impacts on the field, helmets for this sport undergo limited testing for their performance upon low velocity impacts. The accumulation of subconcussive impacts has been associated with functional and microstructural changes in the brains of male athletes (Mainwaring et al., 2018), and in some cases has been associated with the development of neurodegenerative disease (Russell et al., 2021). In our study, we found that at different impact locations, the relative performance of existing helmets often changed according to the impact velocity, with some dramatic changes in relative performance at extreme ends of the tested velocities (e.g., Schutt helmet at FMS location). This suggests that the shock absorbing technologies in the existing helmets may benefit from improvements to ensure a reliable impact response across the wide range of low- and high-velocity impact conditions. Similar to concussive impacts, the liquid helmet we developed offered large reductions in kinematics-based brain injury risk metrics at nearly all subconcussive impact conditions. These reductions could be meaningful for athlete brain health, as the magnitude of head kinematics resulting from subconcussive head impacts has been associated with various indicators of brain structure and function in American football athletes (Joseph et al., 2018; Joseph et al., 2019; Bartsch et al., 2020). The ability of the liquid technology to outperform existing helmet models across such a wide range of impact velocities (1.6 m/s to 9.3 m/s) is likely owed to the ability of liquid-based shock absorbers to scale their force response with impact velocity, rather than displacement as many foam and gas shock absorbers do (Vahid Alizadeh et al., 2021). In this respect, the liquid shock absorbers do not have to be tuned for a single velocity or a narrow range of velocities, and can perform efficiently across many impact velocities. Also similar to the concussive impacts, the only conditions in which the liquid helmet did not outperform all existing helmet models was at the FMCO location. At this impact location, the chinstrap is in tension and a portion of the impact force is transferred to the facemask. The Schutt helmet performed best of all the helmets at the 1.6 m/s FMCO impact condition, suggesting it had a facemask and chinstrap assembly that was optimized for such impacts. Low velocity impacts to the front of the facemask are particularly common for linemen (Bailey et al., 2021). Future designs of liquid shock absorbers and other helmet technologies should not be limited to interior shock absorbers only, and should consider the facemask, chin strap (Spinelli et al., 2018), and other regions of the helmet for potential safety improvements.

Kinematics from the simulated impacts were used as input to the GHBMC FE model of the human head and brain (Mao et al., 2013) in order to calculate brain strains. Similar to the kinematics-based brain injury risk metrics, MPS95 was reduced in nearly all of the concussive and subconcussive impact conditions. These reductions could be clinically meaningful in improving brain health in American football players. Using the GHBMC FE model and a combination of human and primate brain injury data, Wu et al. (2022a) proposed a 50% risk threshold for mild traumatic brain injury of 0.360 MPS95. In our dataset, eleven of the concussive NFL impact conditions resulted in all four of the existing FE helmet models exceeding this threshold. However, the liquid helmet only resulted in an MPS95 value of 0.360 or greater in five of the impact conditions, suggesting that utilizing a liquid helmet could result in a substantial reduction in expected concussions in the NFL helmet test. Using different brain injury criteria and a different helmet test methodology, a previous FE model of a football helmet featuring liquid shock absorbers came to similar conclusions that expected concussions could be dramatically reduced relative to the same open-source FE helmet models (Vahid Alizadeh et al., 2021). For milder head impacts, recent data has proposed that blood brain barrier disruption can occur in sports in the absence of any diagnosed brain injury (O’Keeffe et al., 2020). Although a strain threshold for blood brain barrier disruption based on the GHBMC model has not yet been proposed, Shreiber et al. (1997) proposed a maximum principal strain threshold of 0.188 for blood brain barrier disruption based on FE modeling of a rat brain. In our subconcussive impact tests, the four existing helmet models exceeded an MPS95 value of 0.188 in eight of the impact conditions, while the liquid helmet model only exceeded this value in two of the impact conditions. While these results are encouraging, they should be interpreted with caution, as definitive thresholds for mild traumatic brain injury and blood brain barrier disruption remain elusive, and thresholds based on strain are model-dependent.

Previous research has demonstrated that collapsible liquid shock absorbers are capable of providing a near-ideal force profile that scales with impact velocity (Fanton et al., 2020; Vahid Alizadeh et al., 2022). The liquid shock absorber utilized in this study was inspired by a previous cylindrical shock absorber designed by Vahid Alizadeh et al. (2022), which exerts a reaction force on an impact mass while a fluid contained within the shock absorber is passed through an orifice. However, various material properties, the external dimensions, and orifice size were modified from this previous model for the purposes of integration to a football helmet. Although other designs of liquid shock absorbers, such as “volcano” or telescoping designs, have been shown to yield higher force efficiencies than a cylindrical design in uniaxial, individual unit tests (Fanton et al., 2020; Vahid Alizadeh et al., 2022), it is unclear if these results would translate to a full helmet system. The efficiency of those designs is highly dependent on a precise, variable contact area, which may not be achieved when multiple shock absorbers are engaged in an oblique impact, for example, as would be experienced in a full helmet. Further, one of the purposes of our study was to design a FE model of a helmet that could reasonably be translated to a physical prototype; manufacturing such precise, variable contact areas with a concave side wall out of soft, collapsible fabrics could prove to be difficult.

There are a number of design aspects that should be considered in future FE helmet models featuring liquid shock absorbers. First, while the present study demonstrates the potential improvements afforded by liquid shock absorbers being incorporated throughout an entire helmet, benefits could still be granted by targeting a specific location of a helmet or combining liquid technology with other foams or structures. For example, the American football helmet industry is moving towards position-specific helmets (Lessley et al., 2020); designing a single helmet zone featuring liquid shock absorbers that is targeted towards attenuating frequent impacts unique to a specific position could enable a quicker path to commercialization while maintaining meaningful benefits for athlete safety. Further, our optimization of the orifice area in each liquid shock absorber was homogeneous throughout the entire helmet. Future designs could explore tuning orifice areas and geometries for each specific impact location and could optimize each orifice based on the level of play or player position. An exploration of varying shock absorber geometries and varying materials used for the tripad shells and elastic connections between shells would also be worthwhile. Above all, a liquid shock absorber has yet to be implemented into a physical embodiment of an American football helmet. Any future design directions should consider the product comfort, fit, robustness, and manufacturing costs that are standard and expected in the helmet industry. While we strived to create a helmet architecture that could be recreated in a physical prototype, not all of these aspects of design were heavily considered in the model’s design.

Several limitations exist in the present study. First, time has passed since the FE models of the existing helmets were developed. New helmets have been manufactured that, in their physical embodiments, have outperformed the helmet models that were tested in FE, both in the NFL Helmet Test Protocol and other test methodologies. It remains unknown how much of an improvement our liquid helmet model would have offered if FE models of the latest, top-ranking helmets were available. Further, despite being modeled into the physics of the liquid shock absorber, we did not consider the actual mechanism which would return liquid to the shock absorber after an initial impact. American football helmets are built to withstand multiple impacts, with reconditioning of helmets typically occurring every one or two years. Therefore, a liquid shock absorber design would likely need to meet these needs of the football helmet industry by including a reservoir that contains and returns the fluid to the shock absorber after an impact; without a fluid return mechanism, the performance of the helmet would decrease substantially upon subsequent impacts. Inclusion of a fluid return reservoir may increase the mass of the helmet substantially. Despite this, increased mass has been associated with improved helmet performance in impact testing (McIver et al., 2023), so we do not anticipate that such a modification would have negatively influenced helmet performance. However, it remains unclear how much helmet mass, rather than the physics of the liquid shock absorber, may have contributed to the improved performance of the liquid helmet model relative to the Riddell, Schutt, and Xenith helmet models, which all had lower masses than the liquid helmet. Regardless, the liquid helmet still demonstrated considerable improvements in performance over the VICIS helmet, which had a greater mass than the liquid helmet, indicating that mass alone did not dictate helmet performance. Further, while increasing helmet mass seems to be associated with improved brain safety according to impact tests, it remains unclear how an increase in helmet mass would affect neck injury risk and athlete comfort. Head shapes and sizes vary amongst athletes, and will therefore likely place a different amount of prestress or pressure on the shock absorbers within a helmet. Similar to the other FE models and previous evaluations of these FE models, we did not consider this potentially meaningful aspect of the helmet’s response to impact (Giudice et al., 2020). Finally, the GHMBC model used to calculate brain strains is limited in that it represents only a 50th percentile male. The benefits offered by liquid technology could vary among different brain sizes, which have been shown to yield different levels of injury risk under similar loading conditions (Wu et al., 2022b). The GHBMC is also just one of several currently available FE models of the human head and brain, with some research suggesting that other FE models more accurately represent human brain displacement measurements than the GHBMC (Miller et al., 2017).

Overall, the present study proposes a plausible architecture for a full helmet system featuring liquid shock absorbers. The results of FE simulations with this helmet and other helmet models suggest that liquid technology has the potential to lower the risk of football-related brain injury by attenuating kinematics-based brain injury criteria and brain strains across a wide range of impact velocities. These findings support the future fabrication of helmets featuring liquid shock absorbers to validate these simulation results.

## Data Availability

The raw data supporting the conclusion of this article will be made available by the authors, without undue reservation.

## References

[B1] Bailey A. M. Funk J. R. Crandall J. R. Myers B. S. Arbogast K. B. (2021). Laboratory evaluation of shell add-on products for American football helmets for professional linemen. Ann. Biomed. Eng. 49 (10), 2747–2759. 10.1007/s10439-021-02842-8 34378120

[B2] Bailey A. M. McMurry T. L. Cormier J. M. Funk J. R. Crandall J. R. Mack C. D. (2020a). Comparison of laboratory and on-field performance of American football helmets. Ann. Biomed. Eng. 48, 2531–2541. 10.1007/s10439-020-02627-5 33025320

[B3] Bailey A. M. Sanchez E. J. Park G. Gabler L. F. Funk J. R. Crandall J. R. (2020b). Development and evaluation of a test method for assessing the performance of American football helmets. Ann. Biomed. Eng. 48, 2566–2579. 10.1007/s10439-020-02626-6 33025321

[B4] Bartsch A. J. Hedin D. Alberts J. Benzel E. C. Cruickshank J. Gray R. S. (2020). High energy side and rear American football head impacts cause obvious performance decrement on video. Ann. Biomed. Eng. 48, 2667–2677. 10.1007/s10439-020-02640-8 33111969 PMC7674260

[B5] Baumeister J. Banhart J. Weber M. (1997). Aluminium foams for transport industry. Mater. Des. 18 (4-6), 217–220. 10.1016/s0261-3069(97)00050-2

[B6] Broshek D. K. De Marco A. P. Freeman J. R. (2015). A review of post-concussion syndrome and psychological factors associated with concussion. Brain Inj. 29 (2), 228–237. 10.3109/02699052.2014.974674 25383595

[B54] Bustamante M. C. Bruneau D. Barker J. B. Gierczycka D. Coralles M. A. Cronin D. S. (2019). Component-level finite element model and validation for a modern American football helmet. J. Dyn. Behav. Mater. 5, 117–131.

[B7] Cecchi N. J. Domel A. G. Liu Y. Rice E. Lu R. Zhan X. (2021). Identifying factors associated with head impact kinematics and brain strain in high school American football via instrumented mouthguards. Ann. Biomed. Eng. 49, 2814–2826. 10.1007/s10439-021-02853-5 34549342 PMC8906650

[B8] Choi G. B. Smith E. P. Duma S. M. Rowson S. Campolettano E. Kelley M. E. (2022). Head impact exposure in youth and collegiate American football. Ann. Biomed. Eng. 50 (11), 1488–1497. 10.1007/s10439-022-02974-5 35507229 PMC10081156

[B9] Collins M. Lovell M. R. Iverson G. L. Ide T. Maroon J. (2006). Examining concussion rates and return to play in high school football players wearing newer helmet technology: A three-year prospective cohort study. Neurosurgery 58 (2), 275–286. 10.1227/01.neu.0000200441.92742.46 16462481

[B10] Corrales M. A. Gierczycka D. Barker J. Bruneau D. Bustamante M. C. Cronin D. S. (2020). Validation of a football helmet finite element model and quantification of impact energy distribution. Ann. Biomed. Eng. 48, 121–132. 10.1007/s10439-019-02359-1 31549326

[B11] Davenport E. M. Apkarian K. Whitlow C. T. Urban J. E. Jensen J. H. Szuch E. (2016). Abnormalities in diffusional kurtosis metrics related to head impact exposure in a season of high school varsity football. J. Neurotrauma 33 (23), 2133–2146. 10.1089/neu.2015.4267 27042763 PMC5124736

[B12] Decker W. Baker A. Ye X. Brown P. Stitzel J. Gayzik F. S. (2020). Development and multi-scale validation of a finite element football helmet model. Ann. Biomed. Eng. 48 (1), 258–270. 10.1007/s10439-019-02345-7 31520331 PMC6928099

[B13] Diekfuss J. A. Yuan W. Dudley J. A. DiCesare C. A. Panzer M. B. Talavage T. M. (2021). Evaluation of the effectiveness of newer helmet designs with emergent shell and padding technologies versus older helmet models for preserving white matter following a season of high school football. Ann. Biomed. Eng. 49, 2863–2874. 10.1007/s10439-021-02863-3 34585336

[B14] Dompier T. P. Kerr Z. Y. Marshall S. W. Hainline B. Snook E. M. Hayden R. (2015). Incidence of concussion during practice and games in youth, high school, and collegiate American football players. JAMA Pediatr. 169 (7), 659–665. 10.1001/jamapediatrics.2015.0210 25938704

[B15] Dymek M. Ptak M. Fernandes F. A. (2022). Design and virtual testing of American football helmets–A review. Archives Comput. Methods Eng. 29 (2), 1277–1289. 10.1007/s11831-021-09621-7

[B16] Fanton M. Alizadeh H. V. Domel A. G. Devlin M. Kurt M. Mungal M. G. (2020). Variable area, constant force shock absorption motivated by traumatic brain injury prevention. Smart Mater. Struct. 29 (8), 085023. 10.1088/1361-665x/ab905f

[B17] Foss K. D. B. Yuan W. Diekfuss J. A. Leach J. Meehan W. DiCesare C. A. (2019). Relative head impact exposure and brain white matter alterations after a single season of competitive football: A pilot comparison of youth versus high school football. Clin. J. sport Med. 29 (6), 442–450. 10.1097/jsm.0000000000000753 31688173

[B18] Gabler L. F. Crandall J. R. Panzer M. B. (2019). Development of a second-order system for rapid estimation of maximum brain strain. Ann. Biomed. Eng. 47, 1971–1981. 10.1007/s10439-018-02179-9 30515603

[B19] Giudice J. S. Caudillo A. Mukherjee S. Kong K. Park G. Kent R. (2020). Finite element model of a deformable American football helmet under impact. Ann. Biomed. Eng. 48, 1524–1539. 10.1007/s10439-020-02472-6 32034610

[B20] Giudice J. S. Park G. Kong K. Bailey A. Kent R. Panzer M. B. (2019). Development of open-source dummy and impactor models for the assessment of American football helmet finite element models. Ann. Biomed. Eng. 47, 464–474. 10.1007/s10439-018-02155-3 30341737

[B21] Hoshizaki T. B. Post A. Oeur R. A. Brien S. E. (2014). Current and future concepts in helmet and sports injury prevention. Neurosurgery 75 (4), S136–S148. 10.1227/neu.0000000000000496 25232879

[B22] Joseph J. R. Swallow J. S. Willsey K. Almeida A. A. Lorincz M. T. Fraumann R. K. (2019). Pupillary changes after clinically asymptomatic high-acceleration head impacts in high school football athletes. J. Neurosurg. 133 (6), 1886–1891. 10.3171/2019.7.jns191272 31770721

[B23] Joseph J. R. Swallow J. S. Willsey K. Lapointe A. P. Khalatbari S. Korley F. K. (2018). Elevated markers of brain injury as a result of clinically asymptomatic high-acceleration head impacts in high-school football athletes. J. Neurosurg. 130 (5), 1642–1648. 10.3171/2017.12.jns172386 29966462

[B24] Karton C. Blaine Hoshizaki T. Gilchrist M. D. (2020). A novel repetitive head impact exposure measurement tool differentiates player position in National Football League. Sci. Rep. 10 (1), 1200–1214. 10.1038/s41598-019-54874-9 31992719 PMC6987098

[B25] Kerr Z. Y. Chandran A. Nedimyer A. K. Arakkal A. Pierpoint L. A. Zuckerman S. L. (2019). Concussion incidence and trends in 20 high school sports. Pediatrics 144 (5), e20192180. 10.1542/peds.2019-2180 31615955

[B26] Langlois J. A. Rutland-Brown W. Wald M. M. (2006). The epidemiology and impact of traumatic brain injury: A brief overview. J. head trauma rehabilitation 21 (5), 375–378. 10.1097/00001199-200609000-00001 16983222

[B27] Lessley D. J. Kent R. W. Cormier J. M. Sherwood C. P. Funk J. R. Crandall J. R. (2020). Position-specific circumstances of concussions in the NFL: Toward the development of position-specific helmets. Ann. Biomed. Eng. 48, 2542–2554. 10.1007/s10439-020-02657-z 33078366

[B28] Lessley D. J. Kent R. W. Funk J. R. Sherwood C. P. Cormier J. M. Crandall J. R. (2018). Video analysis of reported concussion events in the National Football League during the 2015-2016 and 2016-2017 seasons. Am. J. sports Med. 46 (14), 3502–3510. 10.1177/0363546518804498 30398897

[B29] Levy M. L. Ozgur B. M. Berry C. Aryan H. E. Apuzzo M. L. (2004). Birth and evolution of the football helmet. Neurosurgery 55 (3), 656–662. 10.1227/01.neu.0000134599.01917.aa 15335433

[B30] Lincoln A. E. Caswell S. V. Almquist J. L. Dunn R. E. Norris J. B. Hinton R. Y. (2011). Trends in concussion incidence in high school sports: A prospective 11-year study. Am. J. sports Med. 39 (5), 958–963. 10.1177/0363546510392326 21278427

[B31] Mack C. D. Solomon G. Covassin T. Theodore N. Cárdenas J. Sills A. (2021). Epidemiology of concussion in the national football League, 2015-2019. Sports health 13 (5), 423–430. 10.1177/19417381211011446 33872087 PMC8404771

[B32] Madhukar A. Ostoja-Starzewski M. (2019). Finite element methods in human head impact simulations: A review. Ann. Biomed. Eng. 47, 1832–1854. 10.1007/s10439-019-02205-4 30693442

[B33] Mainwaring L. Pennock K. M. F. Mylabathula S. Alavie B. Z. (2018). Subconcussive head impacts in sport: A systematic review of the evidence. Int. J. Psychophysiol. 132, 39–54. 10.1016/j.ijpsycho.2018.01.007 29402530

[B34] Manley G. Gardner A. J. Schneider K. J. Guskiewicz K. M. Bailes J. Cantu R. C. (2017). A systematic review of potential long-term effects of sport-related concussion. Br. J. sports Med. 51 (12), 969–977. 10.1136/bjsports-2017-097791 28455362 PMC5466926

[B35] Mao H. Zhang L. Jiang B. Genthikatti V. V. Jin X. Zhu F. (2013). Development of a finite element human head model partially validated with thirty five experimental cases. J. biomechanical Eng. 135 (11), 111002. 10.1115/1.4025101 24065136

[B36] Marks M. E. Holcomb T. D. Pritchard N. S. Miller L. E. Espeland M. A. Miles C. M. (2022). Characterizing exposure to head acceleration events in youth football using an instrumented mouthpiece. Ann. Biomed. Eng. 50, 1–13. 10.1007/s10439-022-03097-7 36274103 PMC9815159

[B37] McCrory P. Meeuwisse W. Dvorak J. Aubry M. Bailes J. Broglio S. (2017). Consensus statement on concussion in sport—The 5th international conference on concussion in sport held in berlin, october 2016. Br. J. sports Med. 51 (11), 838–847. 10.1136/bjsports-2017-097699 28446457

[B38] McIver K. Lee P. Bucherl S. Talavage T. Myer G. Nauman E. (2023). Design considerations for the attenuation of translational and rotational accelerations in American football helmets. J. biomechanical Eng. 145, 061008–061029. 10.1115/1.4056653 PMC10782865 36628996

[B39] Miller L. E. Urban J. E. Stitzel J. D. (2017). Validation performance comparison for finite element models of the human brain. Comput. methods biomechanics Biomed. Eng. 20 (12), 1273–1288. 10.1080/10255842.2017.1340462 PMC5975353 28701050

[B40] National Operating Committee on Standards for Athletic Equipment (NOCSAE) (2021). Standard performance specification for newly manufactured football helmets. NOCSAE Docs, 17m21 (ND)002-17m21.

[B41] Navarro S. M. Sokunbi O. F. Haeberle H. S. Schickendantz M. S. Mont M. A. Figler R. A. (2017). Short-term outcomes following concussion in the NFL: A study of player longevity, performance, and financial loss. Orthop. J. sports Med. 5 (11), 232596711774084. 10.1177/2325967117740847 PMC5714087 29226164

[B42] O'Keeffe E. Kelly E. Liu Y. Giordano C. Wallace E. Hynes M. (2020). Dynamic blood–brain barrier regulation in mild traumatic brain injury. J. neurotrauma 37 (2), 347–356. 10.1089/neu.2019.6483 31702476 PMC10331162

[B43] Rowson S. Duma S. M. (2011). Development of the STAR evaluation system for football helmets: Integrating player head impact exposure and risk of concussion. Ann. Biomed. Eng. 39, 2130–2140. 10.1007/s10439-011-0322-5 21553135

[B44] Rowson S. Duma S. M. Greenwald R. M. Beckwith J. G. Chu J. J. Guskiewicz K. M. (2014). Can helmet design reduce the risk of concussion in football? J. Neurosurg. 120 (4), 919–922. 10.3171/2014.1.jns13916 24484225

[B45] Russell E. R. Mackay D. F. Stewart K. MacLean J. A. Pell J. P. Stewart W. (2021). Association of field position and career length with risk of neurodegenerative disease in male former professional soccer players. JAMA neurol. 78 (9), 1057–1063. 10.1001/jamaneurol.2021.2403 34338724 PMC8329793

[B46] Shreiber D. I. Bain A. C. Meaney D. F. (1997). *In vivo* thresholds for mechanical injury to the blood-brain barrier. SAE Trans. 1997, 3792–3806.

[B47] Spinelli D. J. Plaisted T. A. Wetzel E. D. (2018). Adaptive head impact protection via a rate-activated helmet suspension. Mater. Des. 154, 153–169. 10.1016/j.matdes.2018.04.083

[B48] Vahid Alizadeh H. Fanton M. Camarillo D. B. (2022). Collapsible fluid-filled fabric shock absorber with constant force. J. Intelligent Material Syst. Struct. 33 (4), 590–603. 10.1177/1045389x211023578

[B49] Vahid Alizadeh H. Fanton M. G. Domel A. G. Grant G. Camarillo D. B. (2021). A computational study of liquid shock absorption for prevention of traumatic brain injury. J. biomechanical Eng. 143 (4), 041008. 10.1115/1.4049155 33210108

[B50] Versace J. (1971). A review of the severity index. SAE Technical Paper 710881.

[B51] Viano D. C. Halstead D. (2012). Change in size and impact performance of football helmets from the 1970s to 2010. Ann. Biomed. Eng. 40, 175–184. 10.1007/s10439-011-0395-1 21994057

[B52] Wu T. Rifkin J. A. Rayfield A. C. Anderson E. D. Panzer M. B. Meaney D. F. (2022a). Concussion prone scenarios: A multi-dimensional exploration in impact directions, brain morphology, and network architectures using computational models. Ann. Biomed. Eng. 50 (11), 1423–1436. 10.1007/s10439-022-03085-x 36125606

[B53] Wu T. Sato F. Antona-Makoshi J. Gabler L. F. Giudice J. S. Alshareef A. (2022b). Integrating human and nonhuman primate data to estimate human tolerances for traumatic brain injury. J. biomechanical Eng. 144 (7), 071003. 10.1115/1.4053209 34897386

