# Supplementary material for: Finite element evaluation of an American football helmet featuring liquid shock absorbers for protecting against concussive and subconcussive head impacts
Source: Front Bioeng Biotechnol. 2023 Jun 9;11:1160387. doi: 10.3389/fbioe.2023.1160387 (PMC10287972; doi:10.3389/fbioe.2023.1160387)
Supplement: Supplementary file 1 [file DataSheet1.DOCX]

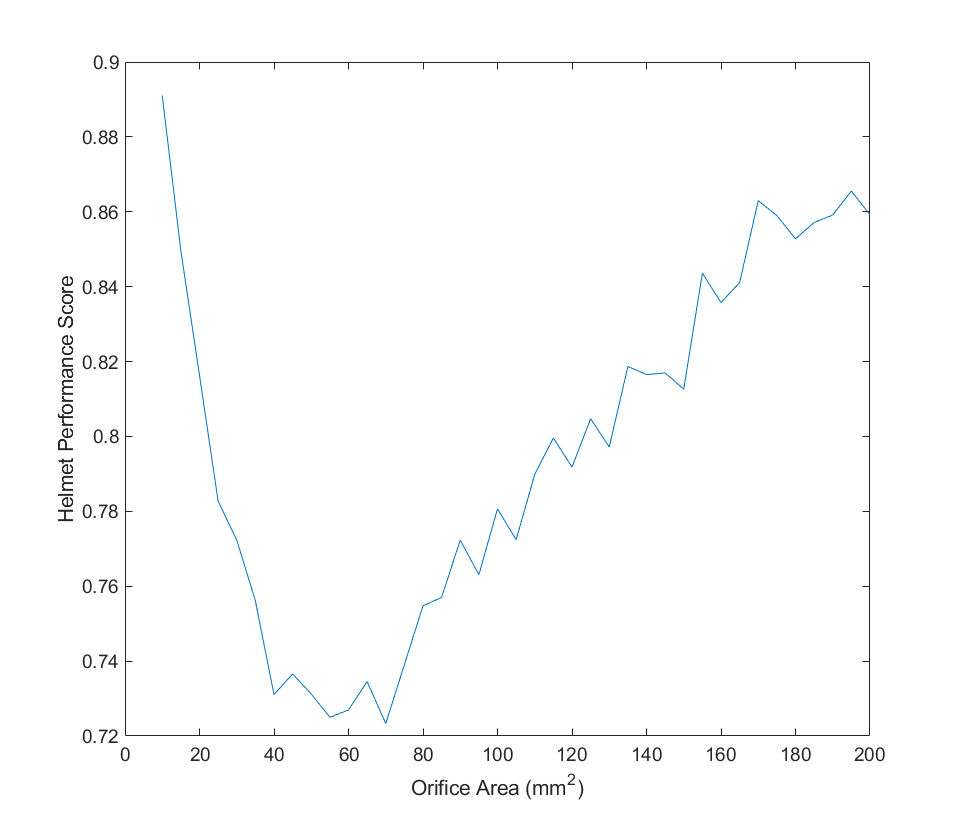


**Supplementary Figure 1**: Helmet Performance Score of the liquid helmet model with cylindrical liquid shock absorbers of varying orifice area. Orifice areas ranging from 10 mm^2^ to 200 mm^2^, in steps of 5 mm^2^, were tested.


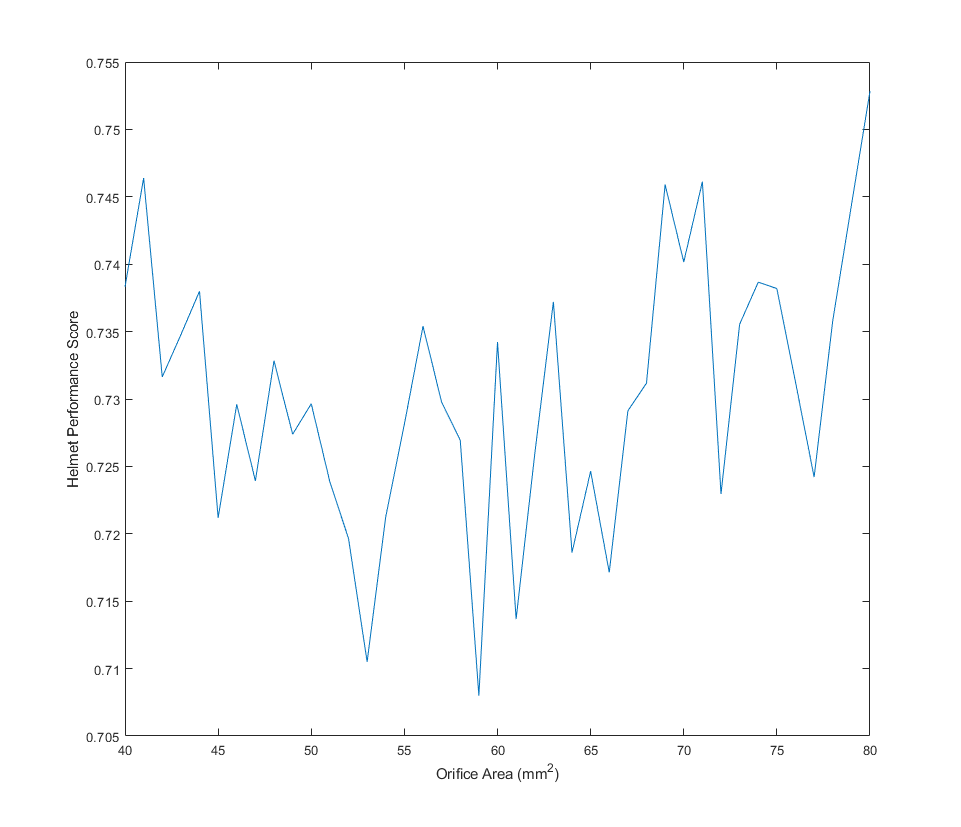
 **Supplementary Figure 2**: Helmet Performance Score of the liquid helmet model with cylindrical liquid shock absorbers of varying orifice area. Orifice areas ranging from 40 mm^2^ to 80 mm^2^, in steps of 1 mm^2^, were tested.





**Supplementary Figure 3:** HIC, DAMAGE, and HARM for concussive velocity impacts. Asterisks denote instances in which impact simulations of the VICIS helmet model erroneously terminated prior to the completion of the impact event. Impact locations: C = Side, D = Oblique Rear, SU = Side Upper, OF = Oblique Front, FMS = Facemask Side, FMCO = Facemask Central Oblique.

**

**

**Supplementary Figure 4:** HIC, DAMAGE, and HARM for subconcussive velocity impacts. Asterisks denote instances in which impact simulations of the VICIS helmet model erroneously terminated prior to the completion of the impact event. Impact locations: C = Side, D = Oblique Rear, SU = Side Upper, OF = Oblique Front, FMS = Facemask Side, FMCO = Facemask Central Oblique.
